# Assessment of cost of innovation versus the value of health gains associated with treatment of chronic hepatitis C in the United States: The quality-adjusted cost of care

**DOI:** 10.1097/MD.0000000000005048

**Published:** 2016-10-14

**Authors:** Zobair M. Younossi, Haesuk Park, Douglas Dieterich, Sammy Saab, Aijaz Ahmed, Stuart C. Gordon

**Affiliations:** a Center For Liver Disease, Department of Medicine, Inova Fairfax Hospital; b Betty and Guy Beatty Center for Integrated Research, Inova Health System, Falls Church, VA; c University of Florida, Gainesville, FL; d Mount Sinai Medical Center, New York, NY; e University of California, Los Angeles, Los Angeles; f Stanford University, Standford, CA; g Henry Ford Hospital, Detroit, MI.

**Keywords:** economic analysis, hepatitis C treatment, quality-adjusted cost of care

## Abstract

**Background:**

New direct-acting antiviral (DAA) therapy has dramatically increased cure rates for patients infected with hepatitis C virus (HCV), but has also substantially raised treatment costs.

**Aim:**

The aim of this analysis was to evaluate the therapeutic benefit and net costs (i.e. efficiency frontier) and the quality-adjusted cost of care associated with the evolution of treatment regimens for patients with HCV genotype 1 in the United States.

**Design:**

A decision-analytic Markov model.

**Data source:**

Published literature and clinical trial data.

**Time horizon:**

Life Time.

**Perspective:**

Third-party payer.

**Intervention:**

This study compared four approved regimens in treatment-naïve genotype 1 chronic hepatitis C patients, including pegylated interferon and ribavirin (PR), first generation triple therapy (boceprevir + PR and telaprevir + PR), second generation triple therapy (sofosbuvir + PR and simeprevir + PR) and all-oral DAA regimens (ledipasvir/sofosbuvir and ombitasvir + paritaprevir/ritonavir + dasabuvir ± ribavirin).

**Outcome measure:**

Quality-adjusted cost of care (QACC). QACC was defined as the increase in treatment cost minus the increase in the patient's quality-adjusted life years (QALYs) when valued at $50,000 per QALY.

**Results:**

All-oral therapy improved the average sustained virologic response (SVR) rate to 96%, thereby offsetting the high drug acquisition cost of $85,714, which resulted in the highest benefit based on the efficiency frontier. Furthermore, while oral therapies increased HCV drug costs by $48,350, associated QALY gains decreased quality-adjusted cost of care by $14,120 compared to dual therapy. When the value of a QALY was varied from $100,000 to $300,000, the quality adjusted cost of care compared to dual therapy ranged from − $21,234 to − $107,861, − $89,007 to − $293,130, − $176,280 to − $500,599 for first generation triple, second generation triple, and all-oral therapies, respectively. Primary efficacy and safety measurements for drug regimens were sourced from clinical trials data rather than a real-world setting. Factors such as individual demographic characteristics, comorbidities and alcohol consumption of the individual patients treated may alter disease progression but were not captured in this analysis.

**Conclusion:**

New DAA treatments provide short-term and long-term clinical and economic value to society.

**Primary funding source:**

Gilead Sciences, Inc.

## Abstract 2

Supplemental Digital Content is available in the text

## 1 Introduction

Hepatitis C virus (HCV) is associated with liver disease and extrahepatic manifestations.^[1–4]^ The financial burden of HCV-related liver disease is estimated to be $6.5 to $13.6 billion,^[5]^ with extrahepatic manifestations adding to the financial burden.^[1–4,6]^

Treatment regimens have rapidly evolved. Historically, a small proportion of patients were able to tolerate the side effects of pegylated interferon (pegIFN) and ribavirin (RBV). Early regimens were also associated with low sustained virologic response (SVR) or “cure” rates.^[7,8]^ When the first-in-class NS3/4A serine protease inhibitors, including boceprevir (BOC) and telaprevir, were added to regimens, SVR rates increased, but tolerability issues remained.^[9,10]^ In addition, real-world data demonstrated lower SVR rates and increased adverse events relative to clinical trials.^[11,12]^ Development of second-generation direct-acting antivirals led to approval of sofosbuvir (SOF) and simeprevir (SMV).^[13,14]^ Although these regimens provided advantages, they still included RBV or both pegIFN and RBV with their associated side effects and impairment of patient-reported outcomes.^[15–17]^ The next advancement led to the approval of interferon-free and RBV-free regimens. These regimens include a single-tablet regimen of ledipasvir (LDV) with SOF and a combination of ombitasvir, dasabuvir, and paritaprevir with ritonavir (OMB/PTV/R + DSV).^[18,19]^ New direct-acting, all-oral antiviral regimens have improved efficacy and tolerability, but are costly.^[20]^ The debate about whether high drug costs for new therapies represent “good value” is ongoing. While cost-effectiveness studies have shown more favorable economic outcomes compared to standard therapies,^[21–23]^ there are still concerns that price may inhibit the potential of therapeutic advances.^[24,25]^

In evaluating therapeutic advances, Lakdawalla et al^[26]^ suggested using “quality-adjusted cost of care” as a practical approach for assessing whether the value of innovative therapies has been worth the cost to society. Given the considerations for HCV, a similar analysis is urgently needed. In conducting this analysis, we applied an approach similar to Lakdawalla et al^[26]^ to establish the quality-adjusted cost of care (long-term effectiveness) of approved HCV treatments. In addition, the therapeutic benefit and net costs of HCV treatments using the efficiency frontier (short-term effectiveness) was assessed. Therefore, the aims of this study were to evaluate the quality-adjusted cost of care and to assess the efficiency frontier with regard to the evolution of treatment strategies for chronic hepatitis C genotype 1 treatment-naïve patients in the United States.

## 2 Materials and methods

### 2.1 Model overview and patient population

A decision-analytic Markov model was developed to estimate health outcomes for antiviral treatment-naïve patients with HCV genotype 1. The model consisted of an initial decision tree in which patients were eligible to receive treatment and a state-transition model to simulate the progression of a 52-year-old genotype 1 patient through HCV natural history and treatment with 1 of 4 treatment strategies. The structure of the model was based on our previously published and validated Markov model.^[23,27]^

**Figure 1:**
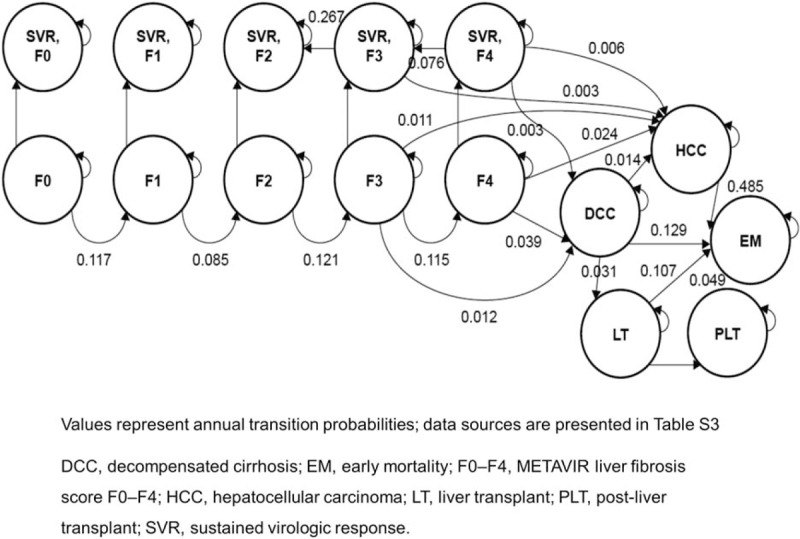
Health state transitions in the model.

Patients infected with HCV genotype 1a represent the majority of the HCV genotype 1 population; therefore, a baseline distribution of 68% for patients with genotype 1a was assumed in the model.^[28,29]^ The chronic phase of the infection was defined based on the METAVIR scoring system: F0—no fibrosis, F1—portal fibrosis without septa, F2—portal fibrosis with few septa, F3—numerous septa without cirrhosis, and F4—with cirrhosis.^[30]^ Patients entered the model at varying stages of liver fibrosis. For this analysis, the baseline distribution was extracted from a recent study: F0—7%, F1—31%, F2—27%, F3—18%, and F4—17%.^[7]^ Upon completion of each regimen, patients were assessed for whether or not they achieved SVR, stratified by fibrosis stage. In the state-transition model, for each 1-year model cycle, patients remained in or transitioned between the following health states: baseline fibrosis stage (F0–F4), SVR stratified by fibrosis score (SVR F0–F4), decompensated cirrhosis (DCC), hepatocellular carcinoma (HCC), liver transplant and post–liver transplant, and death (Fig. 1). Lifetime horizon was modeled until the cohort reached 100 years of age. Outcomes were discounted at 3.0% per year. Subgroup analysis was conducted for patients with cirrhosis and without cirrhosis at the initiation of treatment.

Therapies that have been approved by the Food and Drug Administration or recommended by US professional societies^[20]^ for treatment-naïve patients infected with HCV genotype1 were included. We decided to only include regimens from the past decade that were considered to be previous standards of care. This project did not meet the criteria for human research and was considered as an exempt category.

### 2.2 Clinical inputs

The model incorporated clinical inputs for treatment efficacy, duration and adverse events derived from clinical trials, and published literature for 4 treatment strategies which included all-oral LDV/SOF^[31,32]^ and OMB/PTV/R + DSV ± RBV^[18,33,34]^; second-generation triple SOF + pegIFN and RBV,^[16]^ SMV + pegIFN and RBV^[15,35]^; first-generation triple BOC + pegIFN and RBV,^[10]^ telaprevir (TLV) + pegIFN and RBV^[36]^; and dual (pegIFN and RBV) therapies.^[37]^ The primary efficacy measures were SVR rates, which were assessed 12 weeks after the conclusion of treatment in all regimens. SVR rates and mean treatment duration for each regimen were sourced from appropriate clinical trials and are presented in tables S1 and S2 (“S” tables are included in supplementary material). The proportion of patients experiencing clinically relevant adverse events (significant grade 3/4 hematological adverse events: anemia, neutropenia thrombocytopenia, and hyperbilirubinemia) during clinical trials were incorporated in the model.

### 2.3 Transition probabilities

The annual transition probabilities for the Markov model are presented in Fig. 1 and table S3.^[38–46]^ After completion of treatment, patients who were treated at METAVIR fibrosis score F0 to F2 and who achieved SVR were assumed to maintain SVR and to experience no further disease progression until death. Patients who had a METAVIR fibrosis score of F3 or F4 and who achieved SVR could still experience further disease progression, although at a reduced rate compared with those not achieving SVR. Patients who achieved SVR at METAVIR fibrosis score F3 could progress to HCC but not to DCC, whereas patients who achieved SVR at METAVIR fibrosis score F4 could progress to DCC as well as to HCC.^[40,43]^ In addition, patients who achieved SVR at METAVIR fibrosis score F3 or F4 could experience fibrosis regression with rates derived from studies documenting fibrosis regression in post-SVR patients.^[40,43,47–49]^

Patients who did not achieve SVR were assumed to progress through the natural course of the disease as if untreated and could remain in their current health state or transition to sequential health states in any 1-year model cycle. Patients with a METAVIR fibrosis score of F3 or F4 could additionally progress to advanced liver disease such as DCC or HCC. Those with DCC could transition to HCC or receive a liver transplant, while those with HCC were permitted to transition to liver transplant or death. Probabilities of HCV-related death were taken from published literature on liver-related mortality for the DCC, HCC, and post–liver transplant health states.^[38–46]^ For non-HCV-related causes of death, mortality was based on US general population probabilities by age.^[50]^

### 2.4 Cost of treatments

The model accounted for 4 types of HCV-related cost: drug regimen, treatment monitoring, adverse events, and health state. Calculated drug regimen costs were based on indicated drug dosing, mean clinical trial therapy duration, and unit drug costs that were obtained from Red Book using wholesale acquisition costs as indicated in Table 1.^[51]^ To estimate growth in costs of treating HCV infection, the weighted average drug costs of treatments assuming equal market shares were calculated for the 4 treatment strategies. Weekly monitoring costs varied by treatment regimen and cirrhosis status, calculated according to specified monitoring resource use based on the Resource-Based Relative Values Scale (table S4). Monitoring costs were then aggregated from the weekly totals and treatment duration. Adverse event costs were estimated based on the incidence of each event, and the pharmacy costs and office visits associated with their management. The proportion of patients experiencing clinically relevant adverse events during the clinical trials were incorporated in the model. Pharmacy costs were based on drug treatment algorithms and the wholesale acquisition costs. All adverse events were assumed to necessitate 1 office visit seeing both a doctor and a nurse. For each health state, the model included inpatient, outpatient, emergency department, ambulatory, and pharmacy costs. Costs were stratified by health state and resource type and were estimated based on a mean of 2 previous studies.^[52,53]^ All costs were in 2015 US dollars and, where necessary, were inflated using the medical care component of the consumer price index.^[54]^

**Table 1:**
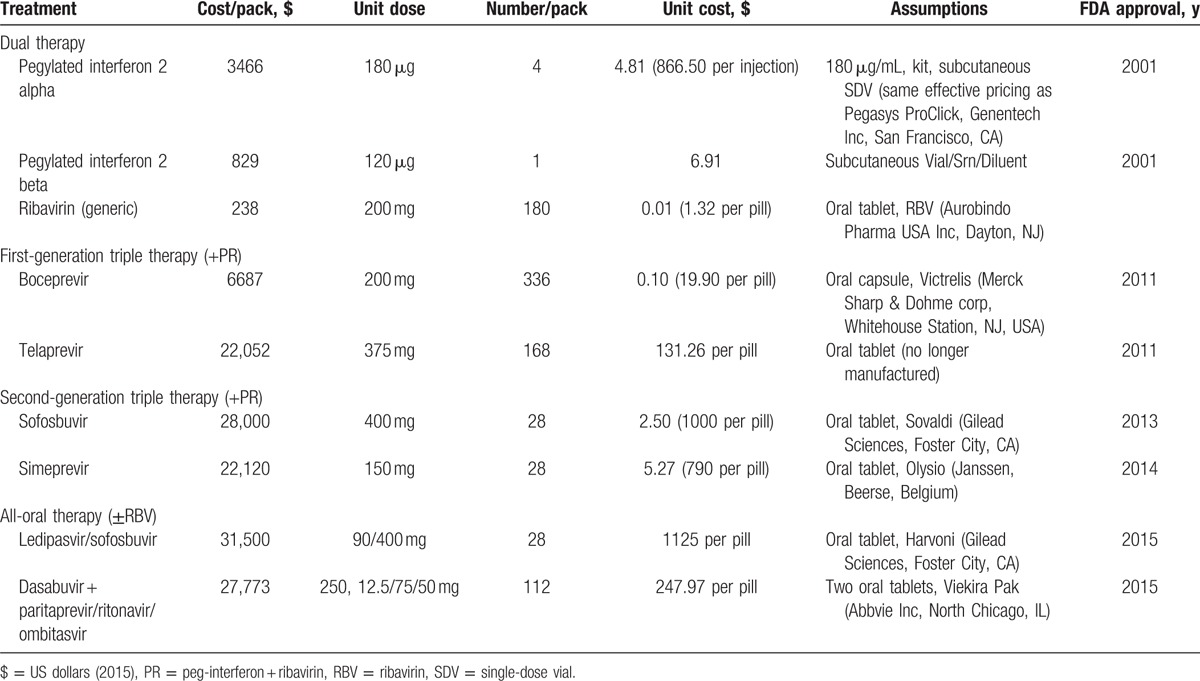
Changes in standard of care for HCV and drug acquisition costs (source: RedBook 2015).

### 2.5 Utility values

Each health state in the model was assigned a utility value that ranged between 1 (perfect health) and 0 (death) to reflect the quality of life of patients in that state; this value was sourced from clinical trial data, when available, as well as published literature^[44,55–58]^ (Table 2). Utility data for LDV/SOF were sourced from a recent study examining the change in health-related quality of life as observed during the ION-1, ION-2, and ION-3 trials.^[55]^ The study examined the changes in the quality of life of patients treated with SOF-based regimens, stratified by pegIFN and RBV (PR), RBV-only, and PR-free regimens. Utility decrements were assigned to each specific treatment regimen and were applied during the treatment duration to account for the negative impact on quality of life associated with adverse events from treatment. Patients who achieved SVR were assumed to receive a utility increment of 0.05.^[56]^

**Table 2:**
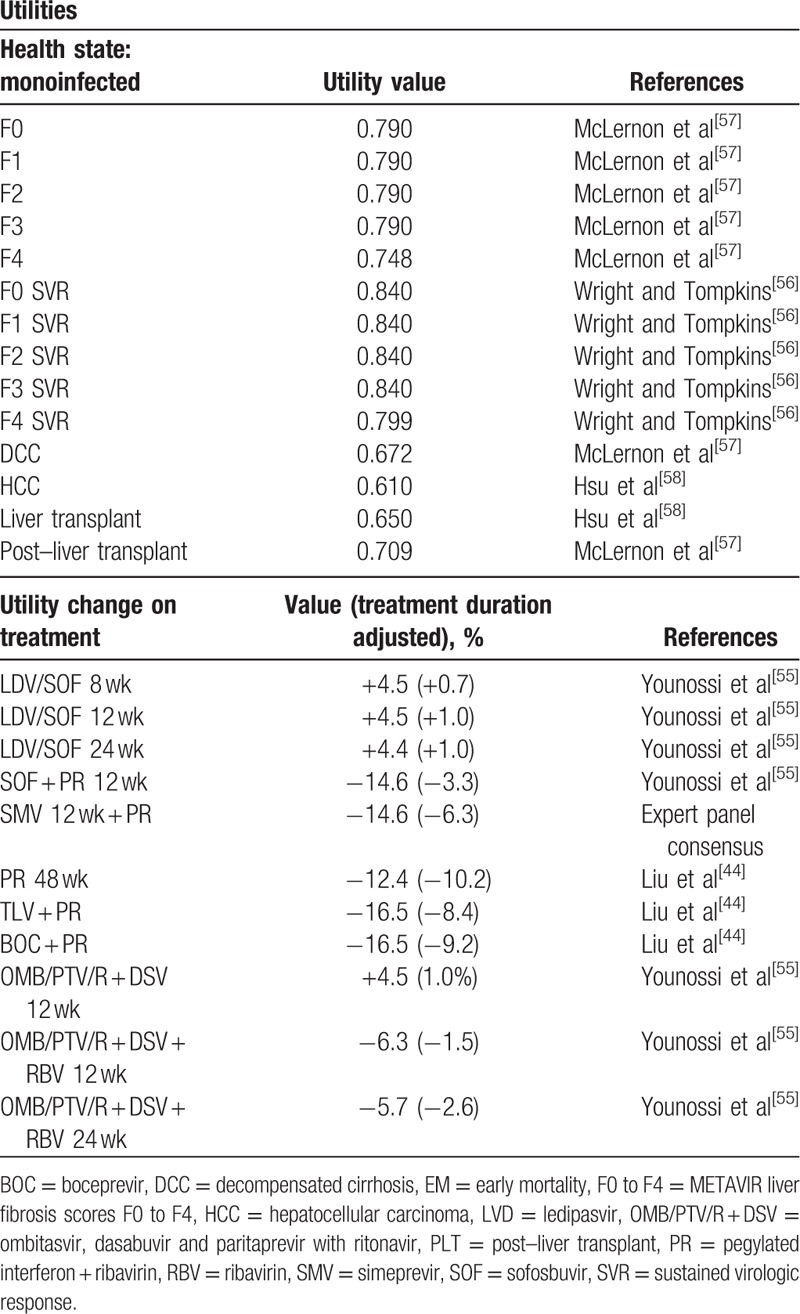
Model utilities.

### 2.6 Efficiency frontier

The efficiency frontier was defined as the ratio of increase in effectiveness (i.e., SVR) and increase in cost.^[59]^ SVR was used to measure treatment effectiveness because SVR is the surrogate marker for HCV cure and the most important clinical parameter determining the success of antiviral therapy. We defined the 2 efficiency frontiers with dual therapy and first-generation triple therapy; and first- and second-generation triple therapies to determine whether the price of all-oral therapy was reasonable (i.e., more efficient than previous standards of care). If all-oral therapy is above the efficiency frontier, this indicates improved efficiency, whereas new drugs being positioned below the efficiency frontier suggest a lower efficiency.^[60]^

### 2.7 Quality-adjusted cost of care

The quality-adjusted cost of care was defined as cost growth net of growth in the value of health improvements, measured as survival gains multiplied by the value of survival. For this analysis, quality-adjusted life years (QALYs) were used to measure health improvement, which captures both life expectancy and quality of life.^[26]^ For each patient, the Markov model estimated a QALY gained during lifetime horizon following HCV treatment and calculated a weighted average of QALY based on the equal distribution of each treatment strategy.

The net change in quality-adjusted cost of care was defined as the increase in treatment costs minus the increase in patient's expected QALYs multiplied by the value of a QALY, which was assumed to be $50,000 for base-case analysis. Threshold values referred to in the literature range from US $50,000 to US $300,000.^[61]^ We used the lower end of this range for base-case analysis. For calculating quality-adjusted cost of care, we used the following approach: quality-adjusted cost of care = (increase in treatment costs) − (monetized increase in quality-adjusted life years [QALYs], defined as increase in QALYs × value of QALY).

Cost growth with new technology is regarded as justified by the associated value to society when the estimated quality-adjusted cost of care is less than 0 but as not justified when the quality-adjusted cost of care is greater than 0. Dual therapy was considered as baseline therapy, which was compared with 3 newer treatment strategies. The trends in the quality-adjusted cost of care as new treatments were introduced, and the potential impact of different treatment strategies were evaluated. Sensitivity analyses were performed to evaluate the impact of varying parameters on results. We evaluated the trends based not only on total treatment costs during lifetime horizon, but also HCV drug costs because new drug costs are key factors driving up healthcare costs. One-way sensitivity analyses were performed using the value of QALY, which ranged from $100,000 to $300,000.

## 3 Results

### 3.1 Efficiency frontier

**Figure 2:**
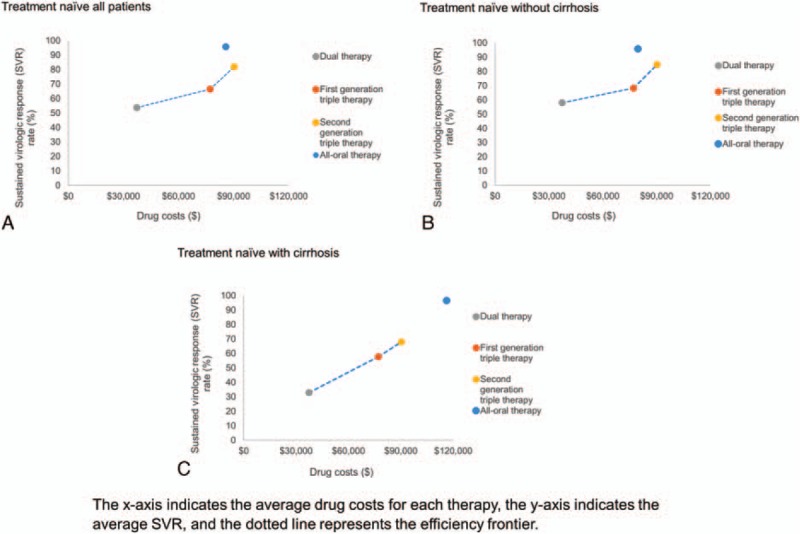
Efficiency frontier of HCV treatment strategies. (A) Treatment naïve all patients, (B) treatment naïve without cirrhosis, and (C) treatment naïve with cirrhosis.

The average SVR rates were estimated to be 54%, 67%, 82%, and 95%, and average drug costs were $37,364, $77,191, $90,292, and $85,714 (Table 3) for dual, first-generation triple, second-generation triple, and all-oral therapies, respectively. Figure 2 illustrates the efficiency frontier for different treatment strategies. Overall, all-oral therapy was above and to the left of the efficiency frontiers suggesting that the price of all-oral therapy was more efficient than any prior treatment strategy (Fig. 2A). In the subgroup analysis for patients without cirrhosis, results show that all-oral therapy was more effective and cheaper than second-generation triple therapy, which was the last technology on the efficiency frontier before all-oral therapy was introduced (Fig. 2B). In patients with cirrhosis, all-oral therapy was more effective but more costly than second-generation triple therapy (Fig. 2C).

**Table 3:**
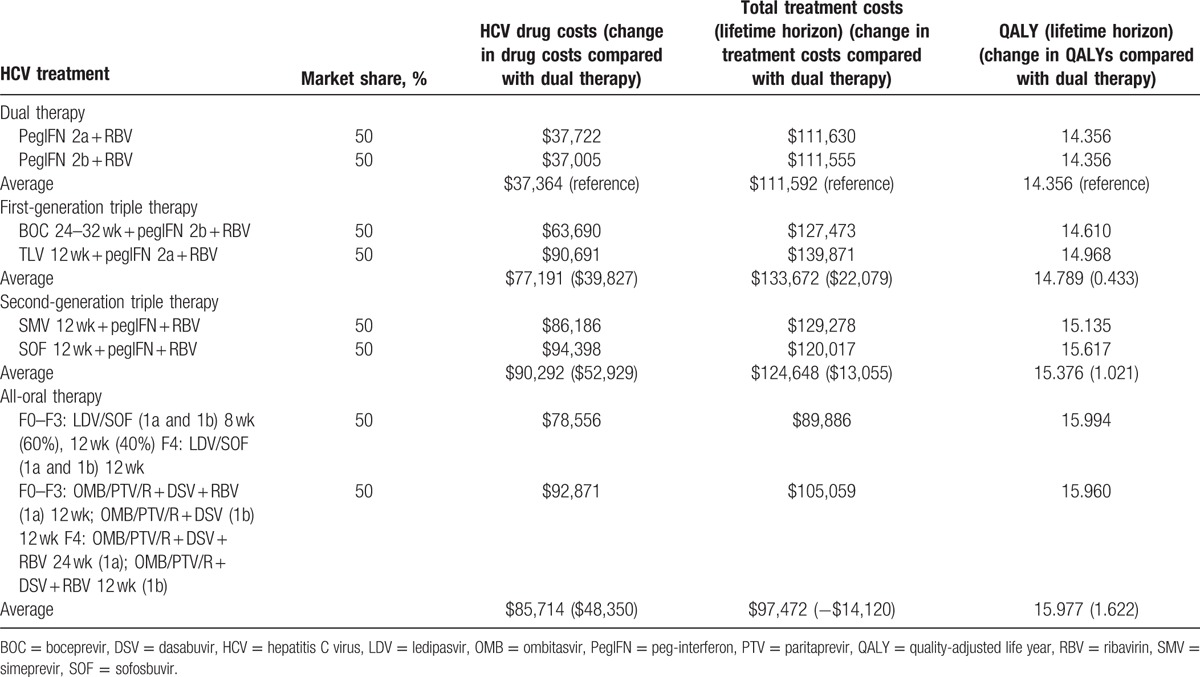
HCV drug costs, treatment costs, and QALYs gained per patient.

### 3.2 Effect of HCV drugs on quality-adjusted cost of care

Table 3 presents the projected long-term economic and health outcomes in terms of QALYs. Although drug treatment costs for HCV infection have continuously increased, new treatments also continuously improved SVR rates and patient outcomes. Compared to dual therapy, treating patients with first-generation triple, second-generation triple, and all-oral therapies was associated with a gain of 0.433, 1.021, and 1.622 QALYs per patient, respectively. The total lifetime costs increased by $22,079 and $13,055 for first- and second-generation triple therapies, but decreased by $14,120 for all-oral therapy.

**Figure 3:**
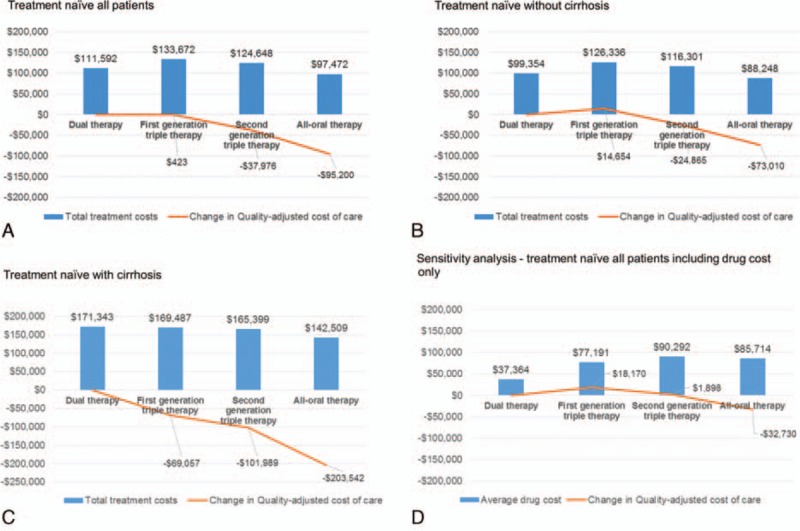
Trends in cost of treatment and quality-adjusted cost of care for HCV. (A) Treatment naïve all patients, (B) treatment naïve without cirrhosis, (C) treatment naïve with cirrhosis, and (D) sensitivity analysis.

The trends in cost of treatment and quality-adjusted cost of care for HCV are presented in Fig. 3. Compared to dual therapy, the average treatment cost increased by $22,079 after first-generation triple therapy was approved, whereas the average QALY improved slightly by 0.433, which resulted in the quality-adjusted cost of care increasing by $423 per person based on a willingness to pay of $50,000 for the value of a QALY. The average total treatment cost for second-generation triple therapy increased by $13,055 per person, and the QALYs increased by 1.021 per person compared to dual therapy. With these estimates, the quality-adjusted cost of care for second-generation triple therapy was reduced to $37,976. The average total treatment cost for all-oral therapy decreased by $14,120, whereas the QALYs increased by 1.622 per person compared to dual therapy, which is valued at $81,080 at the $50,000 per QALY threshold. The estimated quality-adjusted cost of care was calculated at −$95,200, which indicated that offsetting health benefits lowered overall quality-adjusted cost of care by $95,200 for all-oral regimens (due to rounding, calculated results may differ slightly from the exact results).

In the subgroup analysis in patients with and without cirrhosis, the quality-adjusted costs of care were $83,711 (first-generation triple therapy), $77,125 (second-generation triple therapy), and $130,532 (all-oral therapy) lower in patients who initiated treatment at the cirrhotic stage compared with those whose treatment was initiated at the precirrhotic stage (Fig. 3A–C).

### 3.3 Sensitivity analysis

Because of the significant decreases in long-term quality-adjusted costs of care with second-generation triple therapy and all-oral therapy, we also conducted analysis that focused on HCV drug treatment, which is the most important driver of both cost growth and health outcome improvement. Compared to dual therapy, drug costs increased by $39,827, $52,929, and $48,350, whereas health improved by 0.433, 1.021, and 1.622 QALYs for first-generation triple, second-generation triple, and all-oral therapies, respectively (Table 3). Using a willingness-to-pay threshold of $50,000 per QALY, the quality-adjusted costs of care were $18,170, $1898, and −$32,730 for first-generation triple, second-generation triple, and all-oral therapies, respectively, as compared to dual therapy (Fig. 3D). When the value of a QALY ranged from $100,000 to $300,000, the quality-adjusted cost of care compared to dual therapy ranged from −$21,234 to −$107,861, −$89,007 to −$293,130, −$176,280 to −$500,599 for first-generation triple, second-generation triple, and all-oral therapies, respectively, as compared to dual therapy (figure S1).

## 4 Discussion

The quality-adjusted cost of care metric reformulates health economic analysis in a way to help policy-makers better understand trends in healthcare cost growth.^[26]^ As innovation in HCV treatment is being realized, there is need for timely evaluation using more comprehensive metrics of value (i.e., quality-adjusted cost of care) to access whether society is getting what it pays for. This information would provide policy-makers and payers with a simple and transparent framework for assessing whether HCV drug cost growth has been justified by the associated value to society.

This study evaluated the quality-adjusted cost of care with different treatment strategies for treatment-naïve patients with HCV genotype 1. The result of this study showed that HCV drug prices have been generally increasing with the addition of new agents. However, the total treatment costs decreased with all-oral treatments as compared to second-generation triple therapy. The total lifetime costs also decreased with all-oral treatments compared to any prior treatment strategy because of averted liver-disease costs. The quality-adjusted cost of care for first-generation triple therapy increased by $423 per patient. This was largely due to the fact that prices increased without significant offsetting gains in QALYs. However, the quality-adjusted cost of care for second-generation triple therapy and all-oral therapy fell by $37,976 and $95,200 per patient, respectively. When we conservatively assumed only drug costs without taking downstream cost savings into consideration, our results show that the quality-adjusted cost of care for all-oral therapy led to a reduction by $32,730, whereas the quality-adjusted cost of care for first and second triple therapies increased by $18,072 and $1423 per patient, respectively. These data indicate that for the new regimens, society received more benefits than it paid for. Overall, treating patients with cirrhosis (vs without cirrhosis) resulted in even greater value for money to society because of substantially improved outcomes for patients with cirrhosis.

In addition to long-term health benefits, we assessed the short-term effectiveness of treatment regimens using the efficiency frontier method, which has been used by the German Institute for Quality and Efficiency in Health Care.^[59,60]^ This analysis also found that all-oral therapy showed the highest benefit. Both findings indicate that in the short- and long-term, the all oral direct-acting antiviral regimens are beneficial to society.

A number of cost-effectiveness studies have assessed whether the value of a new HCV drug was worth the additional cost. In these economic analyses, cost-effectiveness was assessed with incremental cost-effectiveness ratios (ICERs) with focus on individual drugs. One of the drawbacks of the ICER is that interpretation of negative ICERs (whether interventions are dominant or dominated) cannot be determined without reference to further information. In contrast, the incremental net benefit (INB) analysis provides an unambiguous decision rule, although it implies knowledge of the threshold value.^[62]^ The quality-adjusted cost of care is a conceptually similar approach to INB analysis, which takes into account both value and treatment costs but incorporates value into measurements of cost growth. In this context, value is measured by what society is willing to pay for an additional year of life gained. This threshold has been controversial without a universally accepted monetary value.^[63]^ There is little doubt that $50,000 per QALY is outdated for economic analyses in the United States.^[64]^ Another threshold is recommended by the World Health Organization^[65]^ which connects the accepted threshold to 2 to 3 times the per capita Gross Domestic Product (GDP) of the country. The World Bank^[66]^ reported the 2014 GDP per capita in the United States to be $54,630. Two to 3 times that would be $109,259 to $163,889. The efficiency frontier concept is an extension of the standard approach of ICERs but provides information that can serve as guidance for decision-makers with regard to a setting based on the determination of the prevailing efficiency.^[60]^ In the analysis reported in this investigation, the cost of drug innovation in HCV seems to be offset by benefits to society.

This study has important policy implications. The current data suggest that availability of effective interferon-free all-oral regimens will lead to major changes in the management of HCV and have the potential to greatly affect morbidity and mortality.^[23,67]^ With these new regimens, many barriers to treatment of HCV have largely been overcome as regimens have ease of administration, short duration of treatment, and minimal side effects so that most patients qualify for therapy.^[67]^ However, accessibility to these highly effective drugs remains a barrier because high upfront drug costs might force payers to consider these immediate costs as a higher priority than longer term public health and economic benefits of curing HCV.^[23,67]^ Our findings can provide policy-makers and stakeholders with information to help determine whether the additional costs incurred to cover these new medications provide good value to patients and society.

There are a few potential limitations of this analysis. Primary efficacy and safety measurements for drug regimens were sourced from clinical trials data, which represent a controlled rather than a real-world setting. This may contribute uncertainty in the predicted SVR rates. The model projected the course of liver disease for a cohort of patients based on estimates of natural disease progression data from the literature. It is possible that factors such as individual demographic characteristics, comorbidities, and alcohol consumption of the individual patients treated may alter disease progression,^[68–73]^ which would not be captured in this analysis. However, to estimate transition probabilities, we used either nationally representative, recent data that controlled for covariates or previously used data for HCV models.

In the recent past, HCV treatment took a major step forward with the introduction of all-oral therapies but the rising cost of treatment remains a challenge for patients and payers. This analysis has demonstrated that all-oral therapy offers improved price efficiency with regard to SVR compared to prior standards of care. Furthermore, compared to dual therapy, all-oral regimens increased treatment costs but had offsetting long-term health benefits that lowered the overall quality-adjusted cost of care, whereas patients on first- and second-generation triple therapies experienced drug cost rises with limited corresponding benefits.

## Supplementary Material

Supplemental Digital Content
