# Supplementary material for: Assessment of cost of innovation versus the value of health gains associated with treatment of chronic hepatitis C in the United States: The quality-adjusted cost of care
Source: Medicine (Baltimore). 2016 Oct 14;95(41):e5048. doi: 10.1097/MD.0000000000005048 (PMC5072943; doi:10.1097/MD.0000000000005048)

**Table S1**. Model clinical inputs - SVR rates

|  | Summary of SVR assumption |
| --- | --- |
|  | - When SVR rates are available from multiple clinical trials, the data are weighted based on the size of the trial population - When granular population size is not available, the overall population size is evenly distributed for the granular sub-populations in the weighted mean calculation - When granular fibrotic stage SVRs are unavailable from clinical trials, the non-cirrhotic SVR rate applies to the non-cirrhotic fibrotic stages - When SVR rates are reported for some fibrotic stages but combined for others (e.g., SVR rate is reported for F0−F2 and F3 separately whenever fibrotic distribution data are available), a weighted SVR rate is calculated to generate a non-cirrhotic SVR rate; when data are not available, the overall non-cirrhotic SVR rate would assume the less severe fibrotic stage SVR rate |

a) LDV/SOF 8-week regimen ION-3 [32]

| **Treatment-naïve (HCV viral load <6 million copies)** | | | | | |
| --- | --- | --- | --- | --- | --- |
| **HCV genotype 1a** | | | **HCV genotype 1b** | | |
| **Fibrosis stage** | **SVR, %** | **Source** | **Fibrosis stage** | **SVR, %** | **Source** |
| F0 | 90 | ION-3^a^ | F0 | 94 | ION-3^a^ |
| F1 | 96 | ION-3^a^ | F1 | 100^b^ | ION-3^a^ |
| F2 | 92 | ION-3^a^ | F2 | 96 | ION-3^a^ |
| F3 | 95 | ION-3^a^ | F3 | 100 | ION-3^a^ |
| F4 | N/A | Not indicated | F4 | N/A | Not indicated |

^a^Indicated for treatment-naïve, NC, HIV-negative patients with an HCV viral load <6 million copies. Data are from LDV/SOF 8-week patients from ION-3 with an HCV viral load <6 million copies. Equivalent SVRs are assumed in the overall patient population and the <6 million copies population. RR assumptions from the <6 million copies population are then applied between fibrosis stages, and HCV genotype 1a/1b to the total SVRs.

^b^Any values over 100% were capped at 100%.

F0–F4, METAVIR liver fibrosis score F0–F4; HCV, hepatitis C virus; LDV/SOF, ledipasvir/sofosbuvir; NC, non-cirrhotic; RR, relative rate; SVR, sustained virologic response.

b) LDV/SOF 12-week regimen ION-3 [32], ION-1 [31]

| **Treatment-naïve** | | | | | |
| --- | --- | --- | --- | --- | --- |
| **HVC genotype 1a** | | | **HCV genotype 1b** | | |
| **Fibrosis stage** | **SVR, %** | **Source** | **Fibrosis stage** | **SVR, %** | **Source** |
| F0 | 99 | ION-3^a^ | F0 | 100 | ION-3^a^ |
| F1 | 93 | ION-3^a^ | F1 | 97 | ION-3^a^ |
| F2 | 97 | ION-3^a^ | F2 | 100^b^ | ION-3^a^ |
| F3 | 96 | ION-3^a^ | F3 | 100^b^ | ION-3^a^ |
| F4 | 97 | ION-1^b^ | F4 | 97 | ION-1^b^ |

^a^Indicated for treatment-naïve, NC, HIV-negative patients with an HCV viral load >6 million copies. Data are from LDV/SOF 12-week patients in ION-3 with an HCV viral load >6 million copies. RR assumption applied between fibrosis stages, HCV genotype 1a/1b and the total population.

^b^Indicated for treatment-naïve, CC, HIV-negative patients with an HCV viral load >6 million or <6 million copies.

c) OMB/PTV/R+DSV 12 weeks PEARL-III, PEARL-IV [34]

| **Treatment-naïve^a^** | | | | | |
| --- | --- | --- | --- | --- | --- |
| **HCV genotype 1a** | | | **HCV genotype 1b** | | |
| **Fibrosis stage** | **SVR, %** | **Source** | **Fibrosis stage** | **SVR, %** | **Source** |
| F0 | 92 | PEARL-IV | F0 | 100 | PEARL-III |
| F1 | 92 | PEARL-IV | F1 | 100 | PEARL-III |
| F2 | 83 | PEARL-IV | F2 | 100 | PEARL-III |
| F3 | 90 | PEARL-IV | F3 | 90 | PEARL-III |
| F4 | N/A | Not indicated | F4 | N/A | Not indicated |

d) OMB/PTV/R+DSV + RBV 12 week regimen SAPPHIRE-1 [18], PEARL-IV [34]

| **Treatment-naïve^a^** | | | | | |
| --- | --- | --- | --- | --- | --- |
| **HCV genotype 1a** | | | **HCV genotype 1b** | | |
| **Fibrosis stage** | **SVR, %** | **Source** | **Fibrosis stage** | **SVR, %** | **Source** |
| F0 | 96 | SAPPHIRE-1 /PEARL-IV | F0 | 99 | SAPPHIRE-1 /PEARL-IV |
| F1 | 96 | SAPPHIRE-1 /PEARL-IV | F1 | 99 | SAPPHIRE-1 /PEARL-IV |
| F2 | 94 | SAPPHIRE-1 /PEARL-IV | F2 | 99 | SAPPHIRE-1 /PEARL-IV |
| F3 | 95 | SAPPHIRE-1 /PEARL-IV | F3 | 98 | SAPPHIRE-1 /PEARL-IV |
| F4 | 92 | SAPPHIRE-1 /PEARL-IV | F4 | 100 | TURQUOISE-II |

e) OMB/PTV/R+DSV +RBV 24 week regimen TURQUOISE-11 [33]

| **Treatment-naïve^a^** | | | | | |
| --- | --- | --- | --- | --- | --- |
| **HCV genotype 1a** | | | **HCV genotype 1b** | | |
| **Fibrosis stage** | **SVR, %** | **Source** | **Fibrosis stage** | **SVR, %** | **Source** |
| F4 | 95 | TURQUOISE-II | F4 | NA | Not indicated |

f) SOF + PR regimen NEUTRINO [16]

| **Treatment-naïve^a^** | | | | | |
| --- | --- | --- | --- | --- | --- |
| **HCV genotype 1a** | | | **HCV genotype 1b** | | |
| **Fibrosis stage** | **SVR, %** | **Source** | **Fibrosis stage** | **SVR, %** | **Source** |
| F0 | 102 | NEUTRINO^a^ | F0 | 92 | NEUTRINO^a^ |
| F1−F2 | 93 | NEUTRINO^a^ | F1−F2 | 83 | NEUTRINO^a^ |
| F3 | 92 | NEUTRINO^a^ | F3 | 82 | NEUTRINO^a^ |
| F4 | 80 | NEUTRINO^a^ | F4 | 71 | NEUTRINO^a^ |

^a^RR assumptions applied between HCV genotype 1a/1b and the total patient population.

**g) SMV**+ PR regimen QUEST-1 [15], QUEST-2 [35]

| **Treatment-naïve** | | | | | |
| --- | --- | --- | --- | --- | --- |
| **HCV genotype 1a** | | | **HCV genotype 1b** | | |
| **Fibrosis stage** | **SVR, %** | **Source** | **Fibrosis stage** | **SVR, %** | **Source** |
| F0−F2 | 79 | QUEST-1, -2^a^ | F0−F2 | 89 | QUEST-1, -2^a^ |
| F3 | 69 | QUEST-1, -2^a^ | F3 | 78 | QUEST-1, -2^a^ |
| F4 | 56 | QUEST-1, -2^a^ | F4 | 65 | QUEST-1, -2^a^ |

^a^Weighted mean between QUEST-1 and QUEST-2 studies based on population size.

h) BOC + PR regimen SPRINT-2 [10]

| **Treatment-naïve ^a^** | | | | | |
| --- | --- | --- | --- | --- | --- |
| **HCV genotype 1a** | | | **HCV genotype 1b** | | |
| **Fibrosis stage** | **SVR, %** | **Source** | **Fibrosis stage** | **SVR, %** | **Source** |
| F0−F2 | 63 | SPRINT-2^b^ | F0−F2 | 71 | SPRINT-2^b^ |
| F3 | 48 | SPRINT-2^b^ | F3 | 53 | SPRINT-2^b^ |
| F4 | 52 | SPRINT-2^b^ | F4 | 62 | SPRINT-2[_ENREF_80_ENREF_80](#_ENREF_80)[_ENREF_77](#_ENREF_77)^b^ |

^a^BOC 24 weeks + PR (RGT) is indicated for treatment-naïve, HIV-negative patients. BOC 36 weeks + PR (RGT) is indicated for treatment-experienced, HIV-negative relapsers and prior partial responders. BOC 44 weeks + PR (RGT) is indicated for treatment-experienced, HIV-negative prior null responders.

^b^RR applied between HVC genotype 1a/1b and total population.

i) TLV+ PR regimen ADVANCE [36]

| **Treatment-naïve^a^** | | | | | |
| --- | --- | --- | --- | --- | --- |
| **HCV genotype 1a** | | | **HCV genotype 1b** | | |
| **Fibrosis stage** | **SVR, %** | **Source** | **Fibrosis stage** | **SVR, %** | **Source** |
| F0 | 78 | ADVANCE^a^ | F0 | 86 | ADVANCE^a^ |
| F1 | 78 | ADVANCE^a^ | F1 | 86 | ADVANCE^a^ |
| F2 | 72 | ADVANCE^a^ | F2 | 80 | ADVANCE^a^ |
| F3 | 92 | ADVANCE^a^ | F3 | 65 | ADVANCE^a^ |
| F4 | 80 | ADVANCE^a^ | F4 | 66 | ADVANCE^a^ |

^a^RR assumptions applied between HCV genotype 1a/1b and the total patient population.

j) PR regimen [37]

| **Treatment-naïve^a^** | | | | | |
| --- | --- | --- | --- | --- | --- |
| **HCV genotype 1a** | | | **HCV genotype 1b** | | |
| **Fibrosis stage** | **SVR, %** | **Source** | **Fibrosis stage** | **SVR, %** | **Source** |
| F0 | 58 | Bruno et al. | F0 | 58 | Bruno et al. |
| F1 | 58 | Bruno et al. | F1 | 58 | Bruno et al. |
| F2 | 58 | Bruno et al. | F2 | 58 | Bruno et al. |
| F3 | 58 | Bruno et al. | F3 | 58 | Bruno et al. |
| F4 | 33 | Bruno et al. | F4 | 33 | Bruno et al. |

^a^RR assumptions applied between HCV genotype 1a/1b and the total patient population.

**Table S2**. Model clinical inputs – treatment duration

a) LDV/SOF 8-week regimen ION-3 [32]

| **Treatment-naïve** | | | | |
| --- | --- | --- | --- | --- |
| **Mean treatment duration** | **Discontinuation event** | **Time to discontinuation, weeks** | **Proportion of patients, %** | **Source** |
| LDV/SOF:  8.00 weeks | Completed treatment | 8 | 100 | ION-3 |
|  | Discontinued (AEs) | 4 | 0 | ION-3 |
|  | Discontinued (futility reasons) | 8 | 0 | ION-3 |
|  | Discontinued (other) | 8 | 0 | ION-3 |

AE, adverse event; LDV/SOF, ledipasvir/sofosbuvir.

b) LDV/SOF 12-week regimen ION-1 [31], ION-3 [32]

| **Treatment-naïve** | | | | |
| --- | --- | --- | --- | --- |
| **Mean treatment duration** | **Discontinuation event** | **Time to discontinuation, weeks** | **Proportion of patients, %** | **Source** |
| LDV/SOF:  11.93 weeks | Completed treatment | 12 | 98.37 | ION-1, ION-3^a^ |
|  | Discontinued (AEs) | 8 | 0.47 | ION-1, ION-3^a^ |
|  | Discontinued (futility reasons) | 8 | 0 | ION-1, ION-3^a^ |

^a^Proportion of patients is taken as a weighted mean of ION-1 and ION-3 trials as based on population size.

AE, adverse event; LDV/SOF, ledipasvir/sofosbuvir.

c) OMB/PTV/R+DSV 12 weeks PEARL-III, PEARL-IV [34]

| **Treatment-naïve** | | | | |
| --- | --- | --- | --- | --- |
| **Mean treatment duration** | **Discontinuation event** | **Time to discontinuation, weeks** | **Proportion of patients, %** | **Source** |
| OMB/PTV/R+DSV  11.95 weeks | Completed treatment | 12 | 98.8 | PEARL-III, PEARL-IV^a^ |
|  | Discontinued (AEs) | 8 | 0 | PEARL-III, PEARL-IV^a^ |
|  | Discontinued (futility reasons) | 8 | 0 | PEARL-III, PEARL-IV^a^ |
|  | Discontinued (other) | 8 | 0.7 | PEARL-III, PEARL-IV^a^ |

^a^Proportion of patients is taken as a weighted mean of PEARL-III and PEARL-IV trials as based on population size.

AE, adverse event; OMB/PTV/R+DSV , ombitasvir, dasabuvir and paritaprevir with ritonavir

d) OMB/PTV/R+DSV + RBV 12 week regimen SAPPHIRE-1 [8], PEARL-IV [34]

| **Treatment-naïve** | | | | |
| --- | --- | --- | --- | --- |
| **Mean treatment duration** | **Discontinuation event** | **Time to discontinuation, weeks** | **Proportion of patients, %** | **Source** |
| OMB/PTV/R+DSV + RBV 11.97 weeks | Completed treatment | 12 | 99.3 | SAPPHIRE-1 /PEARL-IV ^a^ |
|  | Discontinued (AEs) | 8 | 0.7 | SAPPHIRE-1 /PEARL-IV ^a^ |
|  | Discontinued (futility reasons) | 8 | 0 | SAPPHIRE-1 /PEARL-IV ^a^ |
|  | Discontinued (other) | 8 | 0.0 | SAPPHIRE-1 /PEARL-IV ^a^ |

^a^Proportion of patients is taken as a weighted mean of SAPPHIRE-I and PEARL-IV trials as based on population size.

AE, adverse event; OMB/PTV/R+DSV , ombitasvir, dasabuvir and paritaprevir with ritonavir

e) OMB/PTV/R+DSV +RBV 24 week regimen TURQUOISE-II [33]

| **Treatment-naïve** | | | | |
| --- | --- | --- | --- | --- |
| **Mean treatment duration** | **Discontinuation event** | **Time to discontinuation, weeks** | **Proportion of patients, %** | **Source** |
| OMB/PTV/R+DSV + RBV 23.41 weeks | Completed treatment | 24 | 95 | TURQUOISE-II |
|  | Discontinued (AEs) | 12 | 2 | TURQUOISE-II |
|  | Discontinued (futility reasons) | 12 | 1 | TURQUOISE-II |
|  | Discontinued (other) | 12 | 2 | TURQUOISE-II |

AE, adverse event; OMB/PTV/R+DSV , ombitasvir, dasabuvir and paritaprevir with ritonavir

**f)** SOF + PR regimen NEUTRINO [16]

| **Treatment-naïve** | | | | |
| --- | --- | --- | --- | --- |
| **Mean treatment duration** | **Discontinuation event** | **Time to discontinuation, weeks** | **Proportion of patients, %** | **Source** |
| SOF + PR 11.91 weeks | Completed treatment | 24 | 98 | NEUTRINO |
|  | Discontinued (AEs, futility, or other) | 12 | 2 | NEUTRINO |

^a^RR assumptions applied between HCV genotype 1a/1b and the total patient population.

g) SMV 12 weeks + PR (RGT) regimen QUEST-1 [15], QUEST-2 [35]

| **Treatment-naïve** | | | | |
| --- | --- | --- | --- | --- |
| **Mean treatment duration** | **Discontinuation event** | **Time to discontinuation, weeks** | **Proportion of patients, %** | **Source** |
| SMV:  11.82 weeks | 4 weeks | 4 | 2.30 | QUEST-1, QUEST-2^a^ |
|  | 4–12 weeks | 12 | 3.55 | QUEST-1, QUEST-2^a^ |
| PR: 22.58 weeks | 12–24 weeks | 24 | 90.50 | QUEST-1, QUEST-2^a^ |
|  | All 48 weeks | 48 | 0 | QUEST-1, QUEST-2^a^ |
|  | Discontinued PR | 12 | 3.65 | QUEST-1, QUEST-2^a^ |

^a^Proportion of patients is taken as a weighted mean of QUEST-1 and QUEST-2 (in treatment-naïve, HIV-negative patients only, 24 weeks of PR is indicated by the FDA label; 24 weeks: calculated as number of patients who met RGT criteria; 4 weeks: calculated as number who discontinued before 4 weeks or who did not meet RGT criteria; 12 weeks: all remaining patients were assumed to discontinue at half of the full treatment range) and QUEST-2 trials as based on population size.

h) BOC + PR (RGT) regimen SPRINT-2 [10]

| **Treatment-naïve** | | | | |
| --- | --- | --- | --- | --- |
| **Mean treatment duration** | **Discontinuation event** | **Time to discontinuation** | **Proportion of patients, %** | **Source** |
| BOC:  23.07 weeks | HCV RNA undetected 8-24 weeks | 28 | 44.02 | SPRINT-2 (briefing book)^a^ |
|  | HCV RNA detectable 8-24 weeks | 48 | 16.58 | SPRINT-2 (briefing book)^a^ |
|  | Discontinued (other) | 36 | 5.71 | SPRINT-2 (briefing book)^a^ |
| PR: 29.05 weeks | Discontinued (lead-in) | 4 | 4.89 | SPRINT-2 (briefing book)^a^ |
|  | Completed PR treatment | 6 | 2.17 | SPRINT-2 (briefing book)^a^ |
|  | Discontinued between 8 and 28 weeks | 24 | 26.63 | SPRINT-2 (briefing book)^a^ |

^a^Proportion of patients taken from the SPRINT-2 trial is broken down as follows: 48 weeks, late responders, factoring out discontinuations; 28 weeks, early responders; 36 weeks, patients who discontinued after 28 weeks (assume timing as midpoint); 24 weeks, patients who discontinued between 8 and 28 weeks; 6 weeks, patients who discontinued between 4 and 8 weeks; 4 weeks, patients who discontinued by week 4.

i) TLV+ PR regimen ADVANCE [36]

| **Treatment-naïve** | | | | |
| --- | --- | --- | --- | --- |
| **Mean treatment duration** | **Discontinuation event** | **Time to discontinuation** | **Proportion of patients, %** | **Source** |
| TLV:  12.00 weeks | 4 weeks | 4 | 3.00 | ADVANCE |
|  | 4–12 weeks | 12 | 97.00 | ADVANCE |
| PR: 26.58 weeks | Completed PR treatment 24 weeks | 24 | 54.00 | ADVANCE |
|  | Completed PR treatment 48 weeks | 48 | 20.00 | ADVANCE |
|  | Stopping rule  (up to week4) | 12 | 3.00 | ADVANCE |
|  | Stopping rule (weeks 4-12)  Discontinued | 24  12 | 7.00  16.00 | ADVANCE  ADVANCE |

j) PR regimen [37]

| **Treatment-naïve** | | | | |
| --- | --- | --- | --- | --- |
| **Mean treatment duration** | **Discontinuation event** | **Time to discontinuation** | **Proportion of patients, %** | **Source** |
| PR: 42.78 weeks | Completed treatment | 48 | 78.2 | Bruno et al. |
|  | Discontinued treatment (AEs) | 24 | 13.3 | Bruno et al. |
|  | Discontinued treatment (other) | 24 | 8.5 | Bruno et al. |

**Table S3**. Model transition probabilities

| **Transition probabilities** | |  |
| --- | --- | --- |
| **From–to** | **Value** | **Source** |
| F0–F1 | 0.117 | Thein, et al. 2008 [41] |
| F1–F2 | 0.085 | Thein, et al. 2008 [41] |
| F2–F3 | 0.121 | Thein, et al. 2008 [41] |
| F3–F4 | 0.115 | Thein, et al. 2008 [41] |
| F3–DCC | 0.012 | Dienstag, et al. 2011 [42] |
| F3–HCC | 0.011 | Dienstag, et al. 2011 [42] |
| F3 SVR–DCC | 0 | Expert opinion |
| F3 SVR–HCC | "(RR=0.24)*0.011  = 0.00264" | Dienstag, et al. 2011 [42]  Morgan, et al. 2013 [45] |
| F4–DCC | 0.039 | Dienstag, et al. 2011 [42] |
| F4–HCC | 0.024 | Dienstag, et al. 2011 [42] |
| F4 SVR–DCC | "(RR=0.0857)*0.039  = 0.00334" | Dienstag, et al. 2011 [42] Morgan, et al. 2013 [45] |
| F4 SVR–HCC | "(RR=0.24)* 0.024  = 0.00576" | Morgan, et al. 2013 [45] |
| DCC–HCC | 0.014 | Fattovich, et al. 1997 [39] |
| DCC–LT | 0.031 | Bennett, et al. 1997 [38] |
| DCC–EM | 0.129 | Fattovich, et al. 1997 [39] |
| HCC–EM | 0.485 | Liu, et al. 2012 [44] |
| LT–EM | 0.107 | Razavi, et al. 2013 [46] |
| PLT–EM | 0.049 | Razavi, et al. 2013 [46] |
| F4 SVR–F3 SVR | 0.076 | D'Ambrosio, et al. 2012 [43] |
| F4 SVR–F2 SVR | 0.082 | Maylin, et al. 2008 [40] |
| F3 SVR–F2 SVR | 0.267 | Maylin, et al. 2008 [40] |

Table S4. Cost of healthcare personnel, laboratory test fees, adverse event management

| **Category** | **Event** | **Cost (2015 $)** |
| --- | --- | --- |
| **Health-care personnel, imaging, and laboratory test fees** | Nurse visit (CPT code: 99211) | $20.12 |
|  | Doctor visit (CPT code: 99214) | $108.88 /3 = $36.29 |
|  | Clinical administration (CPT code: 99285) | $177.15 |
|  | HCV screen (RNA) (CPT code: 87522) | $58.29 |
|  | Urea and electrolytes (CPT code: 84520) | $7.03 |
|  | Glucose (CPT code: 82947) | $5.34 |
|  | Full blood count (CPT code: 85025) | $10.58 |
|  | Ferritin (CPT code:82728) | $18.54 |
|  | Blood clotting factors (CPT code:85610) | $5.35 |
|  | Autoantibodies (CPT code: 86038) | $16.45 |
|  | Immunoglobulins (CPT code: 82784) | $12.65 |
|  | Ultrasound scan of liver (CPT code: 76705) | $93.07 |
|  | Chest X-ray (CPT code:71010) | $22.64 |
|  | ECG (CPT code: 93000) | $17.25 |
|  | HCV genotype (CPT code: 87902) | $350.35 |
| **Adverse event management** | Erythropoeitin-a | $892 per 40,000 IU dose |
|  | Blood transfusion | $484 |
|  | Filgrastim | $ 516 per 480 mcg dose (Neupogen) |
|  | Eltrombopag | $ 199 per 50mg dose |

**Figure S1.** Sensitivity analyses.


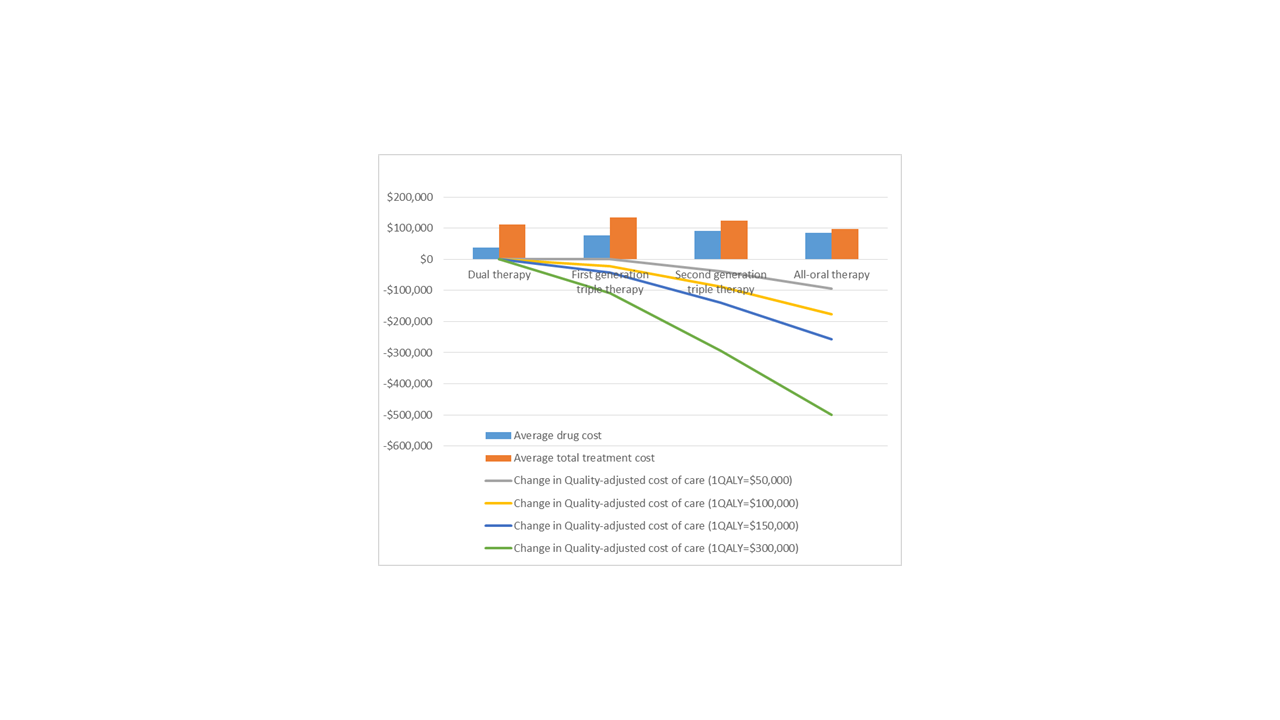

Supplement: Supplemental Digital Content [file medi-95-e5048-s001.doc]
